# Gene and Protein Expression Profile of Selected Molecular Targets Mediating Electrophysiological Function in *Pgc-1α* Deficient Murine Atria

**DOI:** 10.3390/ijms19113450

**Published:** 2018-11-02

**Authors:** Karan R. Chadda, Charlotte E. Edling, Haseeb Valli, Shiraz Ahmad, Christopher L.-H. Huang, Kamalan Jeevaratnam

**Affiliations:** 1 Faculty of Health and Medical Sciences, University of Surrey, Guildford GU2 7AL, UK; krchadda95@gmail.com (K.R.C.); c.edling@surrey.ac.uk (C.E.E.); 2 Physiological Laboratory, University of Cambridge, Downing Street, Cambridge CB2 3EG, UK; haseeb.valli@gmail.com (H.V.); sa416@cam.ac.uk (S.A.); clh11@cam.ac.uk (C.L.-H.H.); 3 Department of Biochemistry, Hopkins Building, University of Cambridge, Cambridge CB2 1QW, UK; 4 School of Medicine, Perdana University-Royal College of Surgeons Ireland, Serdang 43400, Malaysia

**Keywords:** peroxisome proliferator activated receptor-γ (PPARγ) coactivator-1 transcriptional coactivator (Pgc-1), quantitative PCR, ion channels, mitochondria, arrhythmias

## Abstract

Increases in the prevalence of obesity, insulin resistance, and metabolic syndrome has led to the increase of atrial fibrillation (AF) cases in the developed world. These AF risk factors are associated with mitochondrial dysfunction, previously modelled using peroxisome proliferator activated receptor-γ (PPARγ) coactivator-1 (*Pgc-1*)-deficient murine cardiac models. We explored gene and protein expression profiles of selected molecular targets related to electrophysiological function in murine *Pgc-1α^−/−^* atria. qPCR analysis surveyed genes related to Na^+^-K^+^-ATPase, K^+^ conductance, hyperpolarisation-activated cyclic nucleotide-gated (Hcn), Na^+^ channels, Ca^2+^ channels, and indicators for adrenergic and cholinergic receptor modulation. Western blot analysis for molecular targets specific to conduction velocity (Na_v_1.5 channel and gap junctions) was performed. Transcription profiles revealed downregulation of molecules related to Na^+^-K^+^-ATPase transport, Hcn-dependent pacemaker function, Na^+^ channel-dependent action potential activation and propagation, Ca^2+^ current generation, calsequestrin-2 dependent Ca^2+^ homeostasis, and adrenergic α_1D_ dependent protection from hypertrophic change. Na_v_1.5 channel protein expression but not gap junction expression was reduced in *Pgc-1α*^−/−^ atria compared to *WT*. Na_v_1.5 reduction reflects corresponding reduction in its gene expression profile. These changes, as well as the underlying *Pgc-1α^−/−^* alteration, suggest potential pharmacological targets directed towards either upstream PGC-1 signalling mechanisms or downstream ion channel changes.

## 1. Introduction

Cardiac arrhythmias follow breakdown of the ordered action potential (AP) excitation and propagation triggering physiological activity in successive myocardial regions that thereby generates the heartbeat. This, in turn, involves ordered sequences of ion channel activation and inactivation causing AP initiation and recovery. The most common arrhythmia, atrial fibrillation (AF), affects 1–3% of the population in the developed world. Chronic AF increases risks of morbidity, often in the form of stroke and all-cause mortality. Model-based estimates predict substantially increased AF incidences and prevalence in coming decades, resulting in ~9 and ~18 million cases in the United States and Europe, respectively, by 2060 [1].

Both age and acquired metabolic disorders constitute major risk factors for AF. AF thus affects 0.1%, 4%, and 20% of individuals aged <55, 60–70, and >80 years, respectively. Factors such as physical inactivity, obesity, diabetes mellitus and metabolic syndrome, whose occurrence and effects are themselves age-dependent, may explain ~60% of current upward trends in AF incidence [1]. Observational and experimental evidence in turn associate both ageing and metabolic disorder with mitochondrial dysfunction and impaired oxidative capacity [2]. Age-associated mitochondrial DNA damage and compromised respiratory chain function have been demonstrated in a range of human and other mammalian species [3]. Increased mitochondrial dysfunction and defective electron transport chain complex 1 occurs in obese mice on high fat diets [4]. Mitochondrial dysfunction, whether acute or chronic, is known in turn to promote cardiac arrhythmogenesis [5]. The latter is the typical result of altered expression or properties in ion channels underlying electrophysiological activity, or fibrotic or cardiomyopathic change influencing cardiomyocyte or cardiac tissue structure. The latter structural changes have also been reported in experimental diabetes [6] and metabolic syndrome [7].

Mitochondrial function is strongly influenced by members of the peroxisome proliferator activated receptor-γ (PPARγ) coactivator-1 transcriptional coactivator (*Pgc-1*) family. PPARγ coactivators (PGCs) are abundant in oxidative tissues, including cardiac and skeletal muscle, and brown adipose tissue [8]. Modifications in these have been used to examine pathological changes associated with energetic deficiencies. Their expression is impaired in obesity, insulin resistance, type 2 diabetes mellitus (T2DM), and first-degree relatives of diabetic patients [9]. Increasing gene expression related to fatty acid β-oxidation, the tricarboxylic acid cycle and the electron transport chain [10] allows for PGC to regulate mitochondrial biogenesis, mass and function [11]. This, in turn, influences cellular metabolism, and its link to external stimuli driving cellular energy demands [8]. They, thus, constitute potential therapeutic targets. For instance, the PPAR-α agonist fenofibrate, inhibited atrial metabolic remodelling in AF [12,13]. Specific PPARγ ligands such as thiazolidinediones (TZDs) are used in clinical practice to improve insulin sensitivity T2DM [14]. Improvement of cardiac function and reduction in myocardial fibrosis by TZDs have also been previously reported [14]. Finally, rosiglitazone reduces atrial interstitial fibrosis and AF promotion in diabetic rabbits via modulating oxidative stress and inflammation [15]. Of major *Pgc-1α*, *Pgc-1β*, and *Prc* (Pgc-1-related) groups of coactivators [11], *Pgc-1α* appears to have a more dynamic function: *Pgc-1α*, but not *Pgc-1β*, expression is upregulated by physiological stimuli such as fasting, exercise and cold temperatures, thus adapting tissues to high energy demand as opposed to basal mitochondrial function [11].

Experimental models for mitochondrial disorders directed at associated pro-arrhythmic electrophysiological changes are thus central to understanding of their relationships to development of pro-arrhythmic phenotypes. The present experiments utilise mice with a *Pgc-1α^−/−^* genotype to recapitulate features of energetic deficiency. The widespread use of murine models for cardiac electrophysiology research stems from the ability to genetically modify mice to generate targeted disruption modelling human diseases, and thus avoiding the need to use potentially nonspecific pharmacological models [16]. In terms of translatability to human cardiac electrophysiology, murine hearts show similar rapid Na^+^ current (I_Na_) mediated depolarisation phases and transmural AP conduction velocities [17,18,19]. Thus, although murine hearts show anatomical differences, have higher pacing rates and shorter APs compared to human hearts, they have been established to substantially recapitulate human clinical arrhythmic phenotypes [20,21]. Previous studies have successfully validated both *Pgc-1α^−/−^* and *Pgc-1β^−/−^* murine models in the study of mitochondrial dysfunction and arrhythmogenesis [22,23,24,25,26,27,28].

Thus, a higher incidence of extra-systolic provoked atrial arrhythmias, manifesting as atrial tachycardia and ectopic activity, were observed in *Pgc-1β^−/−^* atria [27]. This was attributed to a reduced rate of AP depolarisation (d*V*/d*t*), prolonged AP latencies representing reduced AP conduction and an increased level of myocardial fibrosis [26,27]. Furthermore, at a cellular level, extensive loose patch clamp studies implicated reduced Na^+^ currents but unchanged K^+^ currents as a factor for the previously determined slowed conduction velocity in *Pgc-1β^−/−^* atria [28]. At a systems level, electrocardiographic (ECG) studies in these mice have shown irregular PR, PP and RR intervals with differing P wave morphologies as well as reduced atrioventricular node (AVN) function following adrenergic stimulation [23,24]. Interestingly, murine studies of *Pgc-1α^−/−^* overexpression have shown changes in Ca^2+^ signalling, electrophysiology, and contractile properties [25].

Whilst previous murine electrophysiological studies have shown slowed myocardial conduction velocity as an arrhythmogenic substrate in conditions of mitochondrial dysfunction, the underlying molecular changes remains unexplored. Therefore, the present study firstly uses *Pgc-1α^−/−^* atria in an exploration by quantitative PCR of the gene transcriptional background for changes in genes strategic for the electrophysiological and, therefore, potential arrhythmic phenotypes that may offer possible novel pharmacological targets [29]. Genes were selected and grouped according to the physiological processes underlying excitable activity [16], adapting an approach first applied to rat as opposed to genetically modified mouse hearts [30] comprising: (1) energetically-dependent Na^+^-K^+^-ATPase mediated membrane transport processes generating the ionic gradients driving excitable activity; (2) ion, particularly background K^+^, channels mediating the resting potential; (3) voltage-dependent processes generating both cardiac automaticity and Na^+^ current mediated AP activation; (4) Ca^2+^ homeostatic changes involving both Ca^2+^ channel mediated entry and the subsequent alterations in intracellular Ca^2+^ homeostasis between cellular compartments; (5) electrophysiological recovery from such activity through voltage-dependent K^+^ channel activation; (6) cellular capacity for autonomic modulation through both adrenergic and cholinergic receptor mediated activation of intracellular signalling; (7) a range of cellular and tissue changes ultimately impacting such AP generation and propagation, including inflammatory and tissue fibrotic changes reported in experimental diabetes on earlier occasions. Secondly, this study uses western blots to determine the protein expression levels of Na_v_1.5, Cx40 and Cx43, all critical for determining myocardial conduction velocity. 

## 2. Results

Table 1, Table 2, Table 3, Table 4, Table 5, Table 6, Table 7 and Table 8 summarise results of investigations for independent effects of the *Pgc-1α*^−/−^ genotype on expression of selected murine atrial genes. As indicated in the Methods, samples were obtained by isolating RNA from murine atrial tissue and performing qPCR using ThermoFisher custom Taqman array cards pre-probed with the 60 different genes of potential interest for cardiac function. The samples were divided into two groups, *WT* (*n* = 3) and *Pgc-1α*^−/−^ (*n* = 3). The genetic, fold change, differences between *WT* and *Pgc-1α*^−/−^ atria were calculated using the previously established ΔΔ*C*_t_ method [31]. The statistical results summarised here arise from applications of (a) paired student *t*-tests to assess for differences in expression of functional gene groups between *WT* and *Pgc*-*1α*^−/−^ atria and (b) unpaired student *t*-tests to assess for differences in expression of individual genes between *WT* and *Pgc-1α*^−/−^ atria. The genes explored were subgrouped by function bearing on their mediation of, or maintenance of cellular capacity for electrophysiological function. We stratified the resulting *p* values into the *p* < 0.001, *p* < 0.01, *p* < 0.05, and *p* < 0.10 levels.

### 2.1. Genes Encoding Transport Proteins Mediating Na^+^/K^+^ ATPase Activity

Electrophysiological activity first requires establishment and maintenance of transmembrane ionic gradients that drive its underlying ion fluxes. The gradients are established by energetically dependent membrane transport processes, of which the most fundamental is Na^+^-K^+^ ATPase activity. The resulting Na^+^ and K^+^ transmembrane gradients support cellular osmoregulation, Na^+^-coupled organic and inorganic solute transport and electrical excitability [32]. We accordingly tested transcription activity for the Na^+^-K^+^ ATPase catalytic α_1_ subunit and α_2_ peptide, and accessory β_1_ subunit. These proteins are encoded by *Atpa1*, *Atpa2* and *Atpb1*, respectively. Table 1 summarises mean gene fold changes underlying Na^+^-K^+^ ATPase activity in *Pgc-1α*^−/−^ compared to *WT* atria. It indicates a significant decrease in the level of overall gene expression within the gene group evaluated in *Pgc-1α*^−/−^ compared to *WT* (*p* = 0.01). This appeared to arise from decreased expression of *Atp1a1*, *Atp1a2* and *Atp1b1* in *Pgc-1α*^−/−^ compared to *WT* at the *p* = 0.063, 0.062, and 0.028 levels, respectively, compatible with diminished capacities for ATP-driven Na^+^ and K^+^ transmembrane transport.

### 2.2. Genes Encoding Ion Channel Molecules Mediating Ion Permeabilities Underlying Cardiomyocyte Resting Potential 

The ionic, particularly K^+^, gradients arising from such metabolically dependent transport, sustain a resting potential baseline dependent upon the K^+^ permeabilities of inward rectifier and ATP-sensitive K^+^ and Cl^−^ channels. First, of genes encoding K_ir_2.1 (*Kcnj2*), K_ir_2.2 (*Kcnj12*), and K_ir_2.3 (*Kcnj4*) mediating the I_K1_ K^+^ current, K_ir_2.1 and K_ir_2.2 predominate in human and rabbit ventricle with K_ir_2.3 having less prominent roles in mammalian heart [33]. We surveyed expression of *Kcnj12*, known to encode the ATP-sensitive inward rectifier K^+^ channel 12, K_ir_2.2. Secondly, K_ATP_ channels couple resting potentials to metabolic state through their regulation by intracellular nucleotides [34,35]. This would be relevant to situations with an expected deficiency in intracellular ATP as in the *Pgc-1α*^−/−^ mutant. K_ATP_ channels each comprise four regulatory and four pore-forming subunits. The regulatory subunits are formed by ATP-binding cassette (ABC) transporter subunits of the ATP-sensitive K^+^ channels, members 8 and 9, which also form sulfonylurea receptors, SUR1 and 2 encoded by Abcc8 and Abcc9, respectively. The inwardly rectifying pore-forming K^+^ channel K_ir_6.1 and K_ir_6.2 subunits are encoded by *Kcnj8* and *Kcnj11*, respectively. Cardiac K_ir_6.2/SUR2A K_ATP_ channels may be critical for ischaemic pre-conditioning. Table 2 accordingly surveys *Abcc8* and *Abcc9*, and *Kcnj8* and *Kcnj11*, respectively. 

Thirdly, the G-protein regulated potassium inwardly-rectifying K_ir_3.1 channel mediating I_KACh_ (subfamily J, member 3), encoded by *Kcnj3*, may influence background potentials and exert bradycardic effects in the sinus node [36]. Table 2 also includes *Kcnj5*, encoding the G protein-activated inward rectifier potassium channel 4, GIRK4, whose mutations can cause abnormal aldosterone secretion and hypertension. Finally, we tabulated *Clcn3* encoding the chloride voltage-gated channel 3, important for ischemic preconditioning-induced second-window protection against myocardial infarction [37]. Table 2 summarises our analysis of mean gene-fold changes underlying channels relating to the resting membrane potential in the *Pgc-1α*^−/−^ compared to *WT* atria. The functional group made up of the K^+^ conductance indicated little change in expression levels as a whole and low levels of significance (*p* = 0.66). At the level of individual genes, there were no significant changes in the expression of individual genes between *WT* and *Pgc-1α*^−/−^ to a significance level of *p* < 0.05, with expression levels of only the regulatory *Abcc8* and *Abcc9* subunits of the K_ATP_ channel showing increases with *p* = 0.078 and 0.071, respectively, consistent with little change in the molecular basis for the baseline resting potential. 

### 2.3. Genes Encoding Ion Channels Related to Automaticity and Initiation and Propagation of Excitable Activity

Resting potentials provide the background level from which inward currents drive depolarisation events in pacemaker tissue. Normal pacemaking depends on hyperpolarisation-activated cyclic nucleotide-gated (HCN) channels that drive repetitive action potential activation through generating pacemaker current I_h_ in the sinoatrial node. The HCN family comprises four members that all occur in the heart (HCN1-4) at different expression levels between cardiac regions and species. HCN4 accounts for ~80% of *I*_h_. Of the remaining fractions, HCN1 and HCN2 dominate in rabbits, and in mice and humans, respectively [38]. 

Table 3 summarises mean gene-fold changes underlying channels relating to pacemaker activity and AP initiation in *Pgc-1α*^−/−^ compared to *WT* atria. It demonstrates decreases in the level of gene expression at the functional group level including *Hcn1*, *Hcn3* and *Hcn4* in *Pgc-1α*^−/−^ compared to *WT* to a *p* < 0.01 level. At the individual gene level, there was a decreased *Hcn1* and *Hcn4* expression in *Pgc-1α*^−/−^ compared to *WT* to significance levels of *p* = 0.022 and 0.10, respectively. Pacemaker depolarisation in turn initiates Na^+^ current dependent regenerative processes depolarising the membrane potential thereby resulting in the AP upstroke. The major cardiac Na^+^ channel isoform is Na_v_1.5 [39], but contributions arise from other isoforms in specialized cardiac tissues such as the sino-atrial node [40]. Table 3 demonstrates a marked decrease in cardiac Na_v_1.5 expression to a significance level of *p* = 0.012, strongly suggestive of a potentially pro-arrhythmic compromise in both excitability and its propagation. There was also a downregulation in the atypical Na_v_2.1 encoded by *Scn7a*.

### 2.4. Genes Encoding Ion Channel Molecules Related to Surface Membrane Ca^2+^ Current

AP depolarisation results in surface membrane Ca^2+^ channel activation responsible for both the plateau phase of the cardiac AP and alterations in Ca^2+^ channel homeostasis. The latter arise from release of intracellularly stored sarcoplasmic reticular (SR) Ca^2+^ and both its re-uptake from cytosol back to SR and its exchange transport to the extracellular space. Table 4 surveys gene transcription assessments related to the voltage dependent L-type Ca^2+^ channels Ca_v_1.2 (α_1C_ subunit, encoded by *Cacna1c*), in which loss and gain of function CACNA1C-Q1916R [41] and CACNA1C-L762F mutations [42] result in pro-arrhythmic early and late repolarisation syndromes respectively, as well as Ca_v_1.3 (α_1D_ subunit, encoded by *Cacna1d*). We additionally included the voltage-dependent T-type, Ca_v_3.1 (α_1G_ subunit encoded by *Cacna1g*), and Ca_v_3.2 (α_1H_ subunit encoded by *Cacna1h*), that may function in normal pacemaker activity. Of accessory, nevertheless potentially regulatory subunits, we assayed the Ca^2+^ channel β2 subunit (encoded by *Cacnb2*), and α_2_/δ_1_ (*Cacna2d1*) and α_2_/δ_2_ subunit (*Cacna2d2*) of the voltage-dependent calcium channel complex. *α*_2/_*δ*_2_ is associated with increased peak amplitudes of N-, L- and T-type Ca^2+^ currents in the *Xenopus* oocyte expression system [43]; genetic knockout of α_2_δ_1_ is associated with reduced Ca_V_2.2 levels and reduced Ca^2+^ current densities [44]. Ca^2+^ channels typically comprise complexes of α_1_, α_2_/δ_x_, β, and γ subunits. Table 4 summarises mean gene-fold changes underlying channels related to Ca^2+^ homeostasis in *Pgc-1α*^−/−^ compared to *WT* atria. At the functional group level, changes in the levels of gene expression in *Pgc-1α*^−/−^ relative to *WT* were at *p* = 0.07. This was largely attributable to reduced expression in the regulatory *Cacna2d2* (*p* = 0.005), *Cacna2d1* (*p* = 0.065) subunits and *Cacna1c* and, therefore, Ca_v_1.2 channel (*p* = 0.051) expression. 

### 2.5. Genes Encoding Molecules Related to Intracellular Ca^2+^ Homeostasis

The depolarisation-triggered Ca^2+^ channel mediated entry of Ca^2+^ induces a release of sarcoplasmic reticular (SR) Ca^2+^ central to excitation contraction coupling by the cardiac isoform of the SR ryanodine receptor (RyR2) Ca^2+^ release channel, RyR2. Stress induced polymorphic ventricular tachycardia (PVT) and arrhythmogenic right ventricular dysplasia are conditions associated with RyR2 defects [45]. RyR3 also occurs in cardiac muscle; single nucleotide polymorphisms in this have pharmacogenetic associations with cardiac failure [46]. We assessed transcription in one of the three cardiac SERCA isoforms, *Atp2a2*, the principal cardiac sodium-calcium exchanger (solute carrier family 8 member A1 encoded by *Slc8a1*), and the cardiac isoform of the SR Ca^2+^ binding protein (encoded by *Casq2*) mutations in which cause stress-induced catecholaminergic polymorphic ventricular tachycardia type 2 (CPVT2), characterised by potentially fatal bidirectional ventricular tachycardia. Table 4 indicates that at the functional group level there were small reductions in the level of gene expression in *Pgc-1α*^−/−^ relative to *WT* but only to a *p* = 0.24 significance level. Nevertheless, there were reductions in *Casq2* expression (*p* = 0.05) suggesting reduced SR Ca^2+^ storage capacity, as the only change in molecular background bearing on Ca^2+^ homeostasis. *RyR2* and *RyR3* expression were relatively unchanged.

### 2.6. Genes Encoding Ion Channels Mediating Action Potential Recovery 

The AP recovery phase that restores the transmembrane voltage to the resting potential depends on the action of outward K^+^ currents. These are mediated in murine hearts primarily by the voltage sensitive transient outward current I_to_. This is carried by K_v_1.4 (voltage-gated K^+^ channel subfamily A member 4, encoded by *Kcna4*) and K_v_4.3 (voltage-gated K^+^ channel subfamily D member 3 encoded by *Kcnd3*), both of which are involved in mediating I_to1_ (review: [16]). We also include assays for K_v_11.1 (voltage-gated K^+^ channel subfamily H member 2, K_v_11.1, encoded by *Kcnh2*) mediating the rapid K^+^ current, I_Kr_, in view of its clinical importance. We also analyse the transcription of recently-characterised Ca^2+^-activated K^+^ channels (intermediate/small K^+^ conductance Ca^2+^ -activated K^+^ channel, subfamily N, member 1, K_Ca_2.1, encoded by *Kcnn1*; intermediate/small K^+^ conductance Ca^2+^-activated channel, subfamily N, member 2, K_Ca_2.2, encoded by *Kcnn2*). These are expressed differentially in the atria compared with the ventricles and thought to contribute to the AP repolarisation phase [47]. We include the two-predomain TWIK-related acid-sensitive potassium channel 1 (TASK-1) (K^+^ channel subfamily K member 3; K 2P 3.1, encoded by *Kcnk3*) [48]. Finally, we include the regulatory KCNE1 subunit, encoded by *Kcne1l* in view of its association with some human LQT syndromes. Table 5 indicates that at the functional group level, changes in the level of gene expression in *Pgc-1α*^−/−^ compared to *WT* only showed a significance level of *p* = 0.20. Similarly at the individual gene level, there were no significant changes in the expression of individual genes between *Pgc-1α*^−/−^ and *WT* even to a *p* < 0.1 significance level. These findings suggest an unchanged repolarisation function in the electrical activity of *Pgc-1α*^−/−^ relative to *WT*. 

### 2.7. Genes Encoding Receptors Mediating Cardiac Autonomic Responsiveness

The electrophysiological events above are modulated by autonomic, parasympathetic and sympathetic, inputs which accordingly potentially exert pro or anti-arrhythmic effects following activation of their relevant cardiac receptors. Parasympathetic stimulation has been associated with increased susceptibility to AF, but a canine AF model showed a possible compensatory muscarinic receptor, M_2_, M_3_ and M_4_ mAChR down-regulation [49]. Our current analysis includes *Chrm2*, which encodes the M_2_ muscarinic acetylcholine receptor. The remaining M_1_, M_3_, and M_4_ receptors are thought to occur in neural cells with M_3_ also occurring in smooth muscle, secretory cells and pancreas, rather than cardiac tissue. 

Adrenergic receptors (AR) are similarly G-protein-coupled receptors critical to cardiac physiology. Although accounting for only a minor fraction of cardiac adrenoreceptors, cardiac α_1_-adrenoreceptors mediate important protective and adaptive functions in the heart, through Gq/11 signalling, in particular preventing pathological remodelling in heart failure [50]. We assessed transcription for all three, α_1A_, α_1B_ and α_1D_ (*Adra1a*, *Adra1b*, and *Adra1d*) adrenergic receptor subtypes. This is in addition to our inclusion of both β_1_ and β_2_- adrenergic receptors (*Adrb1* and *Adrb2*), that modify both energy metabolism through cAMP, and stress related PI3K-Akt signalling. Table 6 summarises gene-fold changes underlying cholinergic and adrenergic receptors in *Pgc-1α*^−/−^ compared to *WT* atria. At the level of the entire functional group level there was no significant (*p* = 0.174) change in the level of gene expression between *Pgc-1α^−/−^* and *WT*. At the individual gene level only the decrease in the expression of *Adra1d* encoding the α_1D_ adrenergic receptor in *Pgc-1α^−/−^* compared to *WT* reached a significance level of *p* = 0.035 compatible with otherwise relatively little change in autonomic responsiveness; the latter including even β-adrenergic receptor function. 

### 2.8. Genes Encoding Molecules Mediating Cyclic Nucleotide Signalling 

The cellular capacity for response to autonomic activation depends upon G protein-coupled receptor/adenylyl cyclase/cyclic-3′,5′-adenosine monophosphate (cAMP) signalling. Of the nine isoforms of membrane-bound adenylyl cyclase, our qPCR included *Adcy4* and *Adcy5* encoding the enzymes adenylate cyclase types 4 and 5. Type 5 is one of the two major cardiac isoforms: its transgenic overexpression exacerbates cardiomyopathic changes with chronic catecholamine stimulation. Of eleven major phosphodiesterase subtypes, seven (PDE1, 2, 3, 4, 5, 8, and 9) are expressed in the heart, of which we have assayed transcription of *Pde2a* and *Pde4d* encoding cGMP-dependent 3′,5′-cyclic phosphodiesterase 2A and cAMP-specific 3′,5′-cyclic phosphodiesterase 4D, respectively. Of cAMP targets, we examined *Prkaca* encoding the phosphokinase holoenzyme forming the catalytic subunit α of protein kinase A, and the genes encoding regulatory subunits *Prkar1a* for cAMP-dependent protein kinase type I-α regulatory subunit, and *Prkar2a* and *Prkar2b* for the cAMP-dependent protein kinase type II-α and II-β regulatory subunits respectively [51]. Finally, we assay the major cardiac, calcium/calmodulin-dependent protein kinase, type II-δ, *Camk2d*, which mediates numerous cellular responses to Ca^2+^ signals [52]. Table 7 summarises gene -fold changes bearing on the cAMP pathway in *Pgc-1α^−/−^* compared to *WT* atria. The functional group level showed little difference in gene expression levels comparing *Pgc-1α^−/−^* with *WT* (*p* = 0.45). At the individual gene level, expression of *Adcy4* encoding adenylyl cyclase type 4 but not cardiac type 5 was reduced (*p* = 0.017 and 0.71 respectively), and *Prkar1a* expression increased (*p* = 0.061) in *Pgc-1α^−/−^* compared to *WT*. These findings thus indicate relatively little alteration in gene expression bearing on cyclic nucleotide signalling. 

### 2.9. Genes Encoding Fibrotic Change

Atrial fibrosis has been implicated in pro-arrhythmic atrial conduction disturbances in a range of models including those representing diabetes and age. Their markers examined here include the TGF-β1 isoform (out of TGF-β 1 to 4) for the cytokine transforming growth factor β (TGF-β) [53]. Electrical properties of homo- and heterotypic junctions involving mCx30.2 (encoded by *Gjd3*) may contribute to slow propagation velocity in nodal tissues and directional asymmetry of atrioventricular excitation spread [54]. Finally, we assay mRNA expression of *Col1a1* encoding the major component of type I collagen, the fibrillar collagen found in most connective tissues and the collagen precursor *Col3a1* encoding the collagen type III α1 chain [55]. Results for these genes are summarised in Table 8, and indicate likely insignificant decreases in expression of these (all *p* > 0.1), suggesting that fibrotic changes observed in these circumstances are unlikely to reflect alterations in background gene expression. 

### 2.10. Genes Involving Developmental, Morphological or Other Background Properties 

A final set of genes bearing on a number of nonexcitable physiological phenomena but nevertheless associated with pro- or anti-arrhythmic outcomes included *Tbx3* of the transcriptional repressors *Tbx2* and *Tbx3*, known to affect chamber-specific programs of gene expression that promote differentiation of distinct components of the cardiac conduction system [56]. *Myh6* encodes MHC-α, particularly in human cardiac atria, the major thick filament protein, whose mutations are associated with late-onset hypertrophic cardiomyopathy, atrial septal defects, and sinus node disorder [16]. The nonspecific ion channel Trpc1 conducts both Ca^2+^ and Na^+^ with Ca^2+^ store depletion or activation of the phospholipase C system [57]. Finally, *Nppa* encodes atrial natriuretic peptide, a key autonomic nervous system and ion channel modulator, whose clinical mutations have been implicated in familial atrial fibrillation [58]. A final control assayed expression levels of *Ppargc1a* and *Ppargc1b* respectively encoding Pgc-1α and β, respectively. Table 8 suggests strongly significant alterations in *Tbx3* transcription (*p* < 0.001), but insignificant differences in the remainder tested here (all *p* > 0.1).

### 2.11. Stratification of Gene Expression Differences between Pgc-1α and WT Changes by Magnitude and Significance

Table 9A stratifies particular changes stratified by statistical significance in categories corresponding to probability levels *p* < 0.001, 0.001 < *p* < 0.01, 0.01 < *p* < 0.05, and 0.05 < *p* < 0.1 respectively, in order to identify major features in the transcriptional background that may underlie particular physiological features resulting from the *Pgc-1α^−/−^* genotype. Notable is that all changes with a significance level *p* < 0.05 involved downregulatory although a number stratified between 0.1 < *p* < 0.05 involved upregulatory changes. At a functional group level, such a stratification implicated in descending order of significance: molecules related to pacemaker (*p* = 0.004), Na^+^-K^+^ ATPase activity (*p* = 0.01), Na^+^ channel (*p* = 0.073) and Ca^2+^ channel function (*p* = 0.07), implicating alterations in chronotropic response, energetically dependent aspects of cell ionic homeostasis, activation, and Ca^2+^ homeostasis, respectively. 

**Figure 1:**
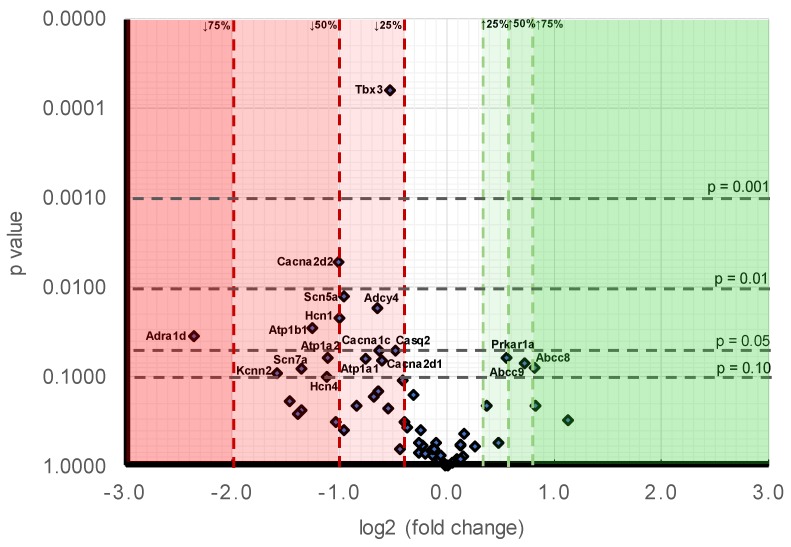
Volcano plot comparing stratified statistical significances in expression levels and mean gene -fold changes in *Pgc-1α^−/−^* compared to *WT* atria for the collection of genes surveyed here. Horizontal stratifications correspond to probability levels *p* < 0.001, 0.001 < *p* < 0.01, 0.01 < *p* < 0.05 and 0.05 < *p* < 0.1 respectively. Ordinates stratify effect sizes indicating decreases/increases of 25%, 50%, and 75% respectively. Points falling within this stratification are labelled with the gene.

Such findings could be compared with their effect sizes (Figure 1) for which the most marked downregulation, reflected in levels of *Pgc-1α^−/−^* normalised to *WT* expression, in descending order of effect size, occurred in Na^+^ channel (0.45 ± 0.06), pacemaker channel (0.45 ± 0.03), Na^+^-K^+^-ATPase (0.49 ± 0.05) and Ca^2+^ channel function (0.72 ± 0.13). All these concordantly yielded *p* < 0.05 in the tests for statistical significance, together giving results compatible with altered expression in molecules subserving metabolically mediated maintenance of ion gradients, SAN automaticity, AP activation and Ca^2+^ homeostasis. This contrasted with both high *p*-values and small effect sizes associated with molecules underlying cAMP signalling (1.10 ± 0.12), concerned with intracellular Ca^2+^ homeostasis (0.90 ± 0.07), K^+^ channels related to AP recovery (0.82 ± 0.12) and to resting potential maintenance (1.09 ± 0.19). 

Figure 1 makes a comparison of the likelihood levels against the alterations of expressions. At the level of individual genes, in order of decreasing significance (Table 9B), the downregulated genes showing differences between *Pgc-1α^−/−^* and *WT* to a probability *p* < 0.01 were *Tbx3* (*p* < 0.001) and *Cacna2d2* (*p* = 0.005). The downregulated genes showing 0.01 < *p* < 0.05 were: *Scn5a* (*p* = 0.012), *Adcy4* (*p* = 0.017), *Hcn1* (*p* = 0.022), *Atp1b1* (*p* = 0.028) and *Adra1d* (*p* = 0.035). The downregulated genes showing 0.05 < *p* < 0.1 were: *Casq2* (*p* = 0.050), *Cacna1c* (*p* = 0.051), *Atp1a2* (*p* = 0.062), *Atp1a1* (*p* = 0.063), *Cacna2d1* (*p* = 0.065), *Scn7a* (*p* = 0.081), *Kcnn2* (*p* = 0.090), and *Hcn4* (*p* = 0.100). Those genes appearing to be upregulated genes all fell within the limits <0.05 *p* < 0.1 were *Prkar1a* (*p* = 0.061), *Abcc9* (*p* = 0.071) and *Abcc8* (*p* = 0.078). A comparison of effect sizes (Table 9B) grouped the magnitudes of such alterations into: expression decreased by >75%: *Adra1d* (0.19 ± 0.08). Expression decreased by between 50–75%: *Kcnn2* (0.33 ± 0.03), *Scn7a* (0.39 ± 0.09), *Atp1b1* (0.42 ± 0.04), *Hcn4* (0.46 ± 0.24) and *Atp1a2* (0.46 ± 0.17). Expression decreased by between 25–50%: *Cacna2d2* (0.50 ± 0.07), *Hcn1* (0.50 ± 0.12), *Scn5a* (0.52 ± 0.04), *Atp1a1* (0.59 ± 0.08), *Adcy4* (0.64 ± 0.05), *Cacna1c* (0.65 ± 0.09), *Cacna2d1* (0.66 ± 0.04), *Tbx3* (0.69 ± 0.02), and *Casq2* (0.72 ± 0.06). Expression increased by between 25–50%: *Pkar1a* (1.47 ± 0.14), *Abcc9* (1.65 ± 0.24) and *Abcc8* (1.77 ± 0.3).

### 2.12. Expression of Proteins Critical for Myocardial Conduction

**Figure 2:**
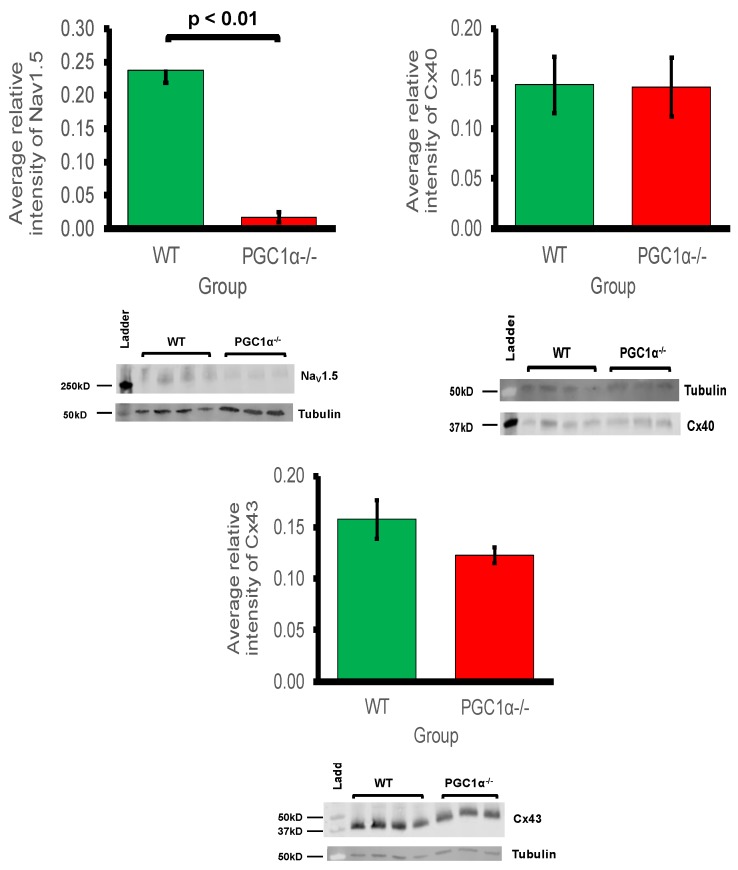
Western blot analysis of atrial Na_v_1.5, Cx40 and Cx43 expression: 3 *WT* and 4 *Pgc-1α^−/−^* atria were used. Representative blots are shown and the housekeeping protein β-tubulin used as a loading control. The primary antibodies used included monoclonal rabbit anti-Na_v_1.5 (1:500); polyclonal goat anti-Cx40 (1:500); polyclonal rabbit anti-Cx43 (1:1000) and polyclonal goat anti-β-tubulin (1:1000). The secondary antibodies included donkey anti-goat IgG antibody in blots for Cx40 and β-tubulin (1:15,000), and donkey anti-rabbit IgG antibody in blots for Na_v_1.5 and Cx43 (1:10,000).

Myocardial conduction velocity is related to the maximum rate of AP depolarisation (dV/dt_max_), which is determined by the current through the Na^+^ channel. Additionally conduction velocity is related to the axial resistance to local circuit currents, largely determined by the conductance of gap junction proteins, typically connexins, as well as membrane capacitances [59]. The main murine connexin isoforms in the atria are Cx40 and Cx43 [60,61]. Figure 2 shows the average relative protein signal intensities of Na_v_1.5, Cx40, and Cx43 in *Pgc-1α^−/−^* and *WT* atria. Thus, compared to *WT* atria, the protein expression of Na_v_1.5 was significantly reduced in *Pgc-1α^−/−^* atria (*p* < 0.01). This is consistent with the qPCR results showing reduced gene expression of *Scn5a*, encoding Na_v_1.5 channel. When considering that peak Na^+^ current is a principal determinant of myocardial conduction velocity, this provides a potential molecular mechanism for the reduced conduction velocity observed in murine models of mitochondrial dysfunction [26,27]. However, the *Pgc-1α^−/−^* genotype was associated with no significant changes in the protein expression levels of Cx40 or Cx43. 

## 3. Discussion

Transcription factors play central roles in atrial remodelling which in turn is a major contributor to development of arrhythmic substrate leading to clinically important conditions such as atrial fibrillation. The present quantitative PCR study screened for changes in genes strategic to cardiac electrophysiological function potentially implicated in atrial arrhythmia that may potentially offer novel pharmacological targets. The studies used as model, the atria of murine hearts deficient in peroxisome proliferator-activated receptor (PPAR)-γ coactivator-1α (*Pgc-1α*). The latter co-activator interacts with the transcription factor and nuclear receptor protein PPAR-α promoting transcription of fatty acid metabolism-related genes. Murine *Pgc-1α^−/−^* hearts thus potentially model a number of the energetic features of atrial fibrillation (AF), the most frequently clinically encountered cardiac arrhythmia. Although there remain significant gaps in our understanding of basic molecular targets in AF pathophysiology [62], recent metabolomic and proteomic studies implicated altered expression in molecules involved in metabolic pathways in human and experimental AF [62,63,64]. AF is accordingly associated with impaired mitochondrial function, decreased ATP production, and redox imbalance with increased reactive oxygen species (ROS) production [65]. The latter potentially injures genes and proteins central to cardiomyocyte function [62].

The present analysis examined expression of genes subclassified into functional groups corresponding to fundamental excitable processes in mouse [16], adapting similar approaches to rat hearts [30]. It surveyed Na^+^-K^+^-ATPase-mediated membrane transport, background K^+^ conductances underlying cardiomyocyte resting potentials, HCN and Na^+^ channels underlying cardiac automaticity and action potential activation and conduction, voltage-dependent Ca^2+^ current activation and intracellular Ca^2+^ homeostatic processes, and action potential recovery through voltage-dependent K^+^ currents. It also examined indicators for adrenergic and cholinergic modulation and their associated intracellular signalling processes, and longer term cellular and tissue changes including tissue fibrotic changes ultimately impacting upon AP generation and propagation.

Modelling studies predict that only large alterations in the related Na^+^/K^+^-ATPase activity would significantly alter cell background membrane potentials [32]. Atrial cardiomyocytes from patients with persistent AF did not show altered Na^+^-K^+^-ATPase current [66]. *Pgc-1α^−/−^* atria nevertheless did show decreased *Atp1a1*, *Atp1a2*, and *Atp1b1* expression encoding regulatory and catalytic Na^+^/K^+^-ATPase subunits compared to levels in *WT*. However, *Pgc-1α^−/−^* atria showed little change in the expression of K^+^ channels underlying resting membrane potential maintenance, suggestive of normal background resting potential properties in *Pgc-1α*^−/−^ relative to *WT*, findings reflected in related *Pgc-1β^−/−^* models [26]. By contrast, of genes encoding ion channels related to automaticity in and initiation and propagation of excitable activity, *Pgc*-*1α*^−/−^ atria showed decreased transcription of pacemaker, *Hcn1* and *Hcn4*, channels relative to *WT*. Such findings are relevant to the chronotropic background and its responses to autonomic activation. The findings recapitulate evidence of impaired canine sinoatrial function, and reduced sino-atrial node *HCN2* and *HCN4* mRNA expression and HCN related current densities in atrial tachypacing studies [67]. Previous ECG studies had demonstrated chronotropic incompetence at the level of the sino-atrial node in *Pgc-1β^−/−^* models of mitochondrial dysfunction following dobutamine challenge [23]. *Pgc-1α^−/−^* atria also showed decreases in cardiac Na_v_1.5 gene expression. This reduction was significant enough to be reflected at the protein level also. Previous studies in murine models of mitochondrial dysfunction have shown reduced Na^+^ current leading to decreased myocardial conduction velocity as a possible arrhythmogenic mechanism [26,27,28]. Thus, reduced Na^+^ current, arising from reduced genetic expression under conditions of mitochondrial dysfunction, is predictive of potentially proarrhythmic reductions in both excitability and AP propagation. This is in common with changes reported in murine models for Brugada Syndrome associated with loss of Na^+^ channel function [27]. Conduction velocity is also determined by gap channel conductance, primarily through connexin isoforms, but this study showed that protein expression levels of Cx40 and Cx43 were not significantly reduced in *Pgc-1α^−/−^* atria. This implies that the primary molecular mechanism for reduced conduction would be reduced Na^+^ channel expression, as opposed to altered gap junction proteins in *Pgc-1α^−/−^* atria.

Atrial cardiomyocytes show reduced L-type Ca^2+^ current, SR Ca^2+^ stores and cellular contractility with metabolic stress [68]. In AF, nuclear factor of activated T-cells (NFAT) is thought to down-regulate Ca_v_1.2 channel α-subunit expression, through the Ca^2+^/calmodulin/calcineurin/NFAT pathway [69,70]. This attenuates L-type Ca^2+^ current and shortens atrial refractory periods [71]. In parallel with this, the qPCR analysis suggested widespread changes in molecules mediating cellular Ca^2+^ homeostasis. Altered transcription in genes encoding ion channel molecules related to membrane Ca^2+^ current manifested as reduced expression of *Cacna1c*, *Cacna2d2*, and *Cacna2d1*. These encode Ca_v_1.2 channels and channel regulatory subunits, respectively. L-type Ca^2+^ channel (Ca_v_1.2) α_1c_ subunit was implicated as an important regulator of reentrant spiral dynamics and as a major component of AF-related electrical remodelling [72]. In addition loss-of-function CACNA1C-Q1916R is thought to contribute to early repolarisation syndrome-related sudden cardiac death [41], and gain of function CACNA1C-L762F with development of LQTS through slower channel inactivation and increased sustained and window current [42]. *α*_2_/*δ*_2_ is associated with increased peak amplitudes of N-, L- and T-type Ca^2+^ currents expressed in *Xenopus* oocytes [43]; genetic knockout of α_2_δ-1 is associated with reduced Ca_V_2.2 levels and reduced Ca^2+^ current densities [44]. Mutations in the regulatory *Cacna2d1* are also associated with cardiac deficiencies, including Brugada and short QT syndromes [73].

Finally, the present observations of reduced *Casq2*-encoded calsequestrin 2 expression, important in SR Ca^2+^ storage capacity, but relatively unchanged *Ryr2* and *Ryr3* expression mediating RyR-mediated SR Ca^2+^ release is compatible with potentially pro-arrhythmic diastolic releases of SR Ca^2+^ associated with either RyR2 [74] or Casq2-mediated [75] atrial pro-arrhythmic changes. The latter are associated with some catecholaminergic polymorphic ventricular tachycardic syndromes. In contrast to the relatively targeted gene functional changes of molecules involved in cellular electrophysiological activation and excitation contraction coupling, we did not observe changes in expression in channels known to be involved in action potential recovery through generation of voltage-dependent K^+^ currents.

Surveys of indicators for adrenergic and cholinergic modulation and their associated intracellular signalling processes demonstrated no significant changes in the cardiac β-adrenergic receptor or atrial parasympathetic receptor, compatible with relatively little change in the transcriptional background to autonomic responsiveness in *Pgc*-*1α*^−/−^ compared to *WT*. Furthermore, changes observed in intracellular autonomic signalling were confined to neuronal adenylyl cyclise type 4, but not the cardiac type 5. Nevertheless, there was decreased expression of *Adra1d*. The latter encodes α_1D_ adrenergic receptors thought to mediate Gq/11 signalling important in protective and adaptive functions against pathologic remodelling in heart failure [50]. Finally, an examination of transcriptional features of molecules involved in longer term cellular and tissue, including fibrotic changes ultimately impacting AP generation and propagation (cf. [55,76]) did not reveal major change.

The findings provide a genomic background for future therapeutic explorations directed at atrial arrhythmias directed at its associated alterations in cellular energetic processes. The therapeutic directions suggested by such findings could suggest explorations of management strategies aimed directly at the ion channels indicated as affected here. The latter could include pacemaker based HCN modulation [67,77,78]. They could also involve pharmacotherapeutic manoeuvres directed at expected alterations in ion channel functions, particularly bearing on the alterations in Ca^2+^ homeostasis suggested here.

Alternatively, possibilities for more upstream therapies are suggested by both clinical studies and experimental results. Studies in rabbit models associated AF with reduced protein expression of sirtuin1, PGC-1α, and PPAR-α. AF patients showed reduced atrial tissue mast cell protease 1 (mCPT-1) and glucose transporter type 4 (GLUT4) protein expression, indicating reduced FA oxidation and glucose transport, compared to control patients in sinus-rhythm. Rabbits in AF showed a similar decrease in these molecules. The PPAR-α agonist fenofibrate then restored expression of mCPT-1 and GLUT4 and activation of the PPAR-α/sirtuin1/PGC-1α pathway, and suppressed AF inducibility [13].

At the level of oxidative function, the bioactive polyphenol resveratrol is associated with diverse benefits including antioxidant cardioprotection, possibly through inducing mitochondrial biogenesis via PGC-1α activation [29]. Resveratrol activates 5’ AMP-activated protein kinase (AMPK)/sirtuin1 signalling [79], modulating cardiac metabolism. In addition, the multifunctional small-molecule resveratrol-derivative, C1 alters function in multiple ion channels mediating ultrarapid and acetylcholine-activated K^+^ currents, as well as Na^+^ currents. It showed antioxidant properties in human and rat atrial cardiomyocytes. It also reduced AF sustainability in atrial tachypaced dogs [80]. Resveratrol also modified remodelling changes associated with AF in a rabbit heart failure (HF) model by activating PI3K/AKT/eNOS signaling and reducing AF susceptibility and triggered activity. This occurred by preventing atrial electrical, contractile, and fibrotic remodelling [81]. It similarly prevented cardiomyopathy and restored cardiac function in mdx mice used to model Duchenne muscular dystrophy [82], possibly through attenuating the expression of the p300 coactivator which is a key contributor to cardiac hypertrophy and fibrosis [82].

The present analysis permits several physiological interpretations relevant to arrhythmogenesis; however this is limited to findings directly related to whole atrial tissue. There is increasing evidence to suggest that the cardiac fibroblast plays an important role in the excitation and propagation of electrical activity. It has been shown that contrary to the classical view, arrhythmogenic activity is not only specific to the cardiac myocyte but is related to heterocellular electrotonic interactions between cardiomyocytes and other cardiac related cells such as the cardiac myofibroblast. Such heterogeneity in electrical properties exist due the varying resting membrane potential of the cardiac myofibroblast relative to that of the cardiac myocyte [83,84]. Additionally, our present work used mice that were of advanced age where some level of fibrotic development may have occurred. It has been shown that in fibrotic hearts, proarrhythmic myofibroblast-cardiomyocyte crosstalk in vitro is mediated by TGF-β_1_ [83]. It is likely that selectively targeting the cardiac myofibroblast may be a useful anti-arrhythmic strategy as well. Given the important role cardiac myofibroblasts play in cardiac electrical properties, future studies should attempt at isolating and differentiating the gene expression and protein profile of native cardiac myofibroblasts and comparing these to corresponding features of native cardiac myocytes. Future studies should be directed at in-vivo electrophysiological interrogation of the *Pgc*-*1α*^−/−^ murine atria including both native cardiac myocytes and cardiac myofibroblasts with particular focus on the identifying functional alterations that may correlate with the molecular findings in the present work. It would be additionally useful for future work studying ventricular gene and protein expression profile in the *Pgc*-*1α*^−/−^ murine to allow comparison between atria and ventricular findings. Such information, whilst potentially not directly comparable due to the variation in tissue electrical properties between atria and ventricles, would allow for elucidation of other mitochondrial dysfunction mediated arrhythmogenic mechanisms.

## 4. Methods

### 4.1. Animals

All experimental protocols were approved under the UK Home Office regulations (Animals (Scientific Procedures) Act 1986 Amendment Regulations 2012) following ethical review by the University of Cambridge Animal Welfare and Ethical Review Body (AWERB) and conducted under a designated project license number PPL70/8726, dated 4 February 2016. All procedures complied with the UK Home Office regulations (Animals (Scientific Procedures) Act 1986). We also followed the Guide for the Care and Use of Laboratory Animals, U.S. National Institutes of Health (NIH Publication No. 85-23, revised 1996). An animal house maintained at 21 °C was used for the mice, with 12-h light/dark cycles. The mice had sterile chow (RM3 Maintenance Diet, SDS, Witham, Essex, UK) and free access to water, bedding and environmental stimuli. Mice were sacrificed by cervical dislocation and no anaesthetic or surgical procedures were required. Wild-type (WT) C57/B6 and *Pgc-1α^−/−^* (The Jackson Laboratory, Bar Harbor, ME, USA) adult mice were bred for the experimental protocols. Mice were bred on a C57/B6 background to avoid possible strain-related confounds. The mice were divided into *WT* and *Pgc-1α^−/−^*, and all were between the ages of 12–24 months old.

### 4.2. Quantitative PCR

In this gene expression study, there were *n* = 3 mice in both the *WT* and *Pgc-1α^−/−^* groups. RNA was extracted from fresh frozen murine atria, stored at −80 °C, with the Qiagen RNeasy mini Plus kit (Qiagen, Manchester, UK)). The atrial tissue was weighed and quickly minced on ice. A third of the tissue, about 30 mg, was used in the next step of the RNA isolation protocol. Tissue pieces were taken from the ice and homogenised in RLT buffer supplemented with beta-mercaptoethanol with a Stuart handheld homogeniser until completely smooth. Genomic DNA was eliminated by centrifugation through a column supplied with the kit prior to extraction of the RNA according to the manufacturer’s protocol. RNA integrity was assessed by using an Agilent bioanalyser (Agilent Technologies, Santa Clara, CA, USA) to obtain RNA integrity numbers (RIN) according to the manufacturer’s protocol. RNA samples with RINs above 8 were used for the study. The RNA was used to prepare cDNA with High Capacity cDNA Reverse Transcription Kit (Applied Biosystems, Waltham, MA, USA) according to the manufacturer’s instructions. The efficiency of the cDNA protocol was tested by preparing the cDNA from a serial dilution of the RNA and then these cDNA samples were run on a qPCR confirming equal efficiency over a range of RNA concentrations. cDNA was also confirmed negative for genomic DNA contamination. Each custom-made card contained 64 pre-validated assays in triplicate with a reaction volume of 1 μL. The cards were run exactly according to instructions specific for the cards. Briefly, the cDNA (100 ng/well) was mixed with 2× Mastermix from Thermo Fisher (Waltham, MA, USA), 100 μL was loaded in each well slot on the cards, the cards were then spun down and sealed and run on a Quant 7 cycler (Applied Biosystems, Waltham, MA, USA). The amplification conditions were: 50 °C for 2 min and 95 °C for 10 min for the initial DNA melting and inactivation of RT reaction, followed by 40 cycles of 95 °C for 15 s and 60 °C for 60 s. Analysis of the Taqman array card data was performed by using the Quant studio software (Applied Biosystems, Waltham, MA, USA) and Microsoft Excel (Microsoft Corporation, Redmond, WA, USA) by calculating fold changes with the ΔC*t* method as previously described [31]. The threshold was set at 0.2 fluorescence units and the baseline range was set to automatic assignment. A combination of HPRT, Gapdh and ActinB were used as reference genes and amplifications were calculated with the regression threshold and baseline subtraction curve fit auto settings with the BioRad CFX manager software. The statistical analysis involved use of paired Student’s *t*-test to compare for differences in expression of functional gene groups and use of unpaired Student’s *t*-test to compare for differences in expression of individual genes between WT and *Pgc*-*1α*^−/−^ atria, for a stratification of values of *p*.

### 4.3. Western Blots

Murine atria were extracted and homogenised in 450µl of lysis buffer (150 mM NaCl, 25 mM tris(hydroxymethyl)aminomethane (tris), pH 7–8, 1% Triton-X100 detergent, 5 mM ethylenediaminetetraacetic acid (EDTA) and Roche^®^ cOmplete™ mini protease inhibitor (Merck KGaA, Darmstadt, Germany)). After a 20-min centrifugation at 12,000 RPM, the clear lysate was obtained and a bicinchoninic acid (BCA) assay performed to assess for protein content (Thermo Scientific Microplate BCA Protein Assay Kit #23252: manufacturer recommended protocol). The samples underwent sodium dodecyl sulphate polyacrylamide gel electrophoresis (SDS-PAGE) using loading buffer (12.8 mL tris, pH 6.8, 3.2 g sodium dodecyl sulphate (SDS), 1.85 g dithiothreitol (DTT), 16 mL 100% glycerol, bromophenol blue, 11.2 mL H_2_O) in the ratio of 3:1 volume of clear lysate to loading buffer. The mixtures were heated at 70 °C for 5 min and loaded into Mini-Protean TGX™ (Bio-Rad, Watford, UK), 4–15% acrylamide gradient, precast gel wells (20 µg for Na_v_1.5 blots and 30 µg for Cx40 and Cx43 blots). Samples were run at 120 V for 30 min, then 250 V for 20 min and compared to β-tubulin as a loading control. Semi-dry transfer of the proteins was carried out onto polyvinylidene fluoride (PVDF) membranes (Immobilon™ PVDF membrane, Merck KGaA, Darmstadt, Germany) using Trans-Blot^®^ Turbo™ kit (BioRad, Watford, UK). The transfer settings were 1.3 A current and 25 V potential for 10 min. The PVDF membranes were then blocked with Odyssey^®^ blocking buffer (Li-Cor Biosciences, Cambridge, UK) for one hour at room temperature, rinsed with PBS-T (0.1% Tween) and incubated with primary antibody diluted in Odyssey^®^ blocking buffer diluted 33% in PBS-T overnight at 4 °C. The primary antibodies used were Na_v_1.5 (Cell Signalling Technology, London, UK), Cx43 (Sigma-Aldrich Company Ltd, Gillingham, UK), Cx40 (Santa Cruz Biotechnology, Dallas, TX, USA) and β-tubulin (Abcam, Cambridge, UK). The membranes were washed three times and then incubated with secondary antibodies diluted in Odyssey^®^ blocking buffer diluted 33% in PBS-T at room temperature for 45 min. Imaging of the blots utilised the Odyssey^®^ Fc imaging system (Li-Cor Biosciences, Cambridge, UK), which measured emission from the secondary antibodies at 600 and 800 nm. Image Studio™ software (Image Studio 4.0, Li-Cor Biosciences, Cambridge, UK) was used to quantify the protein band intensity and subtract the background signal, and then express this relative to the control, β-tubulin, signal. Statistical analysis was performed using the unpaired Student’s *t*-test.

## Figures and Tables

**Table 1:** The mean gene-fold changes underlying Na^+^-K^+^ ATPase activity in *Pgc-1α^−/−^* compared to *WT* atria.

Gene	Protein	Mean WT Fold Change (*n* = 3)	WT SE	Mean *Pgc-1α*^−/−^ Fold Change (*n* = 3)	*Pgc-1α*^−/−^ SE	*t*-Test (Unpaired) *p* Value
*Atp1a1*	Na-K-transporting ATPase subunit α1	1.00	0.14	0.59	0.08	0.063
*Atp1a2*	Na-K-transporting ATPase subunit α2	1.00	0.12	0.46	0.17	0.062
*Atp1b1*	Na-K-transporting ATPase subunit β1	1.00	0.17	0.42	0.04	0.028

**Table 2:** The mean gene-fold changes underlying channels relating to the resting membrane potential in *Pgc-1α^−/−^* compared to *WT* atria.

Gene	Protein	Mean WT Fold Change (*n* = 3)	WT SE	Mean *Pgc-1*α^−/−^ Fold Change (*n* = 3)	*Pgc-1*α^−/−^ SE	*t*-Test (Unpaired) *p* Value
*Kcnj12*	ATP-sensitive inward rectifier K^+^ channel subfamily J member 12, Kir2.2	1.00	0.13	0.98	0.16	0.941
*Abcc8*	ATP-binding cassette (ABC) transporter subunit of ATP-sensitive K^+^ channels, member 8; SUR1	1.00	0.12	1.77	0.30	0.078
*Abcc9*	ATP-binding cassette (ABC) transporter subunit of ATP-sensitive K^+^ channels, member 9; SUR2	1.00	0.11	1.65	0.24	0.071
*Kcnj8*	ATP-sensitive, inwardly-rectifying K^+^ channel subfamily J, member 8, K_ir_6.1	1.00	0.16	0.68	0.15	0.228
*Kcnj11*	ATP-sensitive, inward rectifying K^+^ channel, subfamily J, member 11, K_ir_6.2	1.00	0.44	0.39	0.05	0.239
*Kcnj3*	G-protein regulated inwardly-rectifying K^+^ channel, subfamily J, member 3; K_ir_3.1 (GIRK1)	1.00	0.23	1.04	0.20	0.906
*Kcnj5*	G protein-activated inward rectifier K^+^ channel 4; Kv3.4 (GIRK4)	1.00	0.05	1.09	0.14	0.568
*Clcn3*	voltage-gated Cl- channel 3	1.00	0.05	0.75	0.11	0.110

**Table 3:** The mean gene fold changes underlying channels relating to the initiation of excitable activity in *Pgc-1α^−/−^* compared to *WT* atria.

Gene	Protein	Mean WT Fold Change (*n* = 3)	WT SE	Mean *Pgc-1*α^−/−^ Fold Change (*n* = 3)	*Pgc-1*α^−/−^ SE	*T*-Test (Unpaired) *p* Value
*Hcn1*	Hyperpolarisation activated cyclic nucleotide-gated channel 1	1.00	0.07	0.50	0.12	0.022
*Hcn3*	Hyperpolarisation activated cyclic nucleotide-gated channel 3	1.00	0.45	0.38	0.15	0.262
*Hcn4*	Hyperpolarisation activated cyclic nucleotide-gated channel 4	1.00	0.09	0.46	0.24	0.100
*Scn5a*	Na^+^ voltage-gated channel type 5 subunit α, Na_v_1.5	1.00	0.11	0.52	0.04	0.012
*Scn7a*	Na^+^ voltage-gated channel type 7 subunit α, Na_v_2.1	1.00	0.25	0.39	0.09	0.081

**Table 4:** The mean gene fold changes underlying Ca^2+^ homeostasis in *Pgc-1α^−/−^* compared to *WT* atria.

Gene	Protein	Mean WT Fold Change (*n* = 3)	WT SE	Mean *Pgc-1α^−/−^* Fold Change (*n* = 3)	*Pgc-1α^−/−^* SE	*t*-Test (Unpaired) *p* Value
*Cacna1c*	Ca^2+^ channel, voltage-dependent, L type, α1C subunit (Ca_v_1.2)	1.00	0.09	0.65	0.09	0.051
*Cacna1d*	Ca^2+^ channel, voltage-dependent, L type, α1D subunit (Ca_v_1.3)	1.00	0.39	0.36	0.09	0.189
*Cacna1g*	Ca^2+^ channel, voltage-dependent, T type, α1G subunit (Ca_v_3.1)	1.00	0.16	0.86	0.18	0.581
*Cacna1h*	Ca^2+^ channel, voltage-dependent, T type, α1H subunit (Ca_v_3.2)	1.00	0.17	0.64	0.10	0.146
*Cacnb2*	Ca^2+^ channel, voltage-dependent, L type, β-2 subunit	1.00	0.14	1.39	0.59	0.550
*Cacna2d1*	Ca^2+^ channel, voltage-dependent, α_2_/δ_1_ subunit	1.00	0.13	0.66	0.04	0.065
*Cacna2d2*	Ca^2+^ channel, voltage-dependent, α_2_/δ_2_ subunit	1.00	0.05	0.50	0.07	0.005
*Ryr2*	Ryanodine receptor type 2	1.00	0.19	0.91	0.18	0.753
*Ryr3*	Ryanodine receptor type 3	1.00	0.61	0.99	0.33	0.987
*Atp2a2*	Sarco/endoplasmic reticulum Ca^2+^-ATPases (SERCA)	1.00	0.21	0.77	0.06	0.368
*Slc8a1*	Na^+^-Ca^2+^ exchanger solute carrier family 8 member A1	1.00	0.24	1.11	0.28	0.772
*Casq2*	Calsequestrin 2	1.00	0.08	0.72	0.06	0.050

**Table 5:** The mean gene fold changes underlying channels relating to repolarisation in *Pgc-1α^−/−^* compared to *WT* atria.

Gene	Protein	Mean WT Fold Change (*n* = 3)	WT SE	Mean *Pgc-1α^−/−^* Fold Change (*n* = 3)	*Pgc-1α^−/−^* SE	*t*-Test (Unpaired) *p* Value
*Kcna4*	voltage-gated K^+^ channel subfamily A member 4 (K_v_1.4)	1.00	0.21	1.20	0.28	0.603
*Kcnd3*	voltage-gated K^+^ channel subfamily D member 3 (K_v_4.3)	1.00	0.06	0.94	0.08	0.547
*Kcnh2*	voltage-gated K^+^ channel subfamily H member 2 (K_v_11.1)	1.00	0.19	0.62	0.11	0.168
*Kcnn1*	intermediate/small K^+^ conductance Ca^2+^-activated channel, subfamily N, member 1 (K_Ca_2.1)	1.00	0.05	0.95	0.21	0.845
*Kcnn2*	intermediate/small K^+^ conductance Ca^2+^-activated channel, subfamily N, member 2 (K_Ca_2.2)	1.00	0.30	0.33	0.03	0.090
*Kcnk3*	K^+^ channel subfamily K member 3 (K2P3.1)	1.00	0.27	0.87	0.20	0.719
*Kcne1l*	voltage-gated K^+^ channel subfamily E regulatory subunit 5	1.00	0.13	1.07	0.29	0.841

**Table 6:** The mean gene fold changes underlying adrenergic and cholinergic receptors in *Pgc-1α^−/−^* compared to *WT* atria.

Gene	Protein	Mean WT Fold Change (*n* = 3)	WT SE	Mean *Pgc-1α^−/−^* Fold Change (*n* = 3)	*Pgc-1α^−/−^* SE	*t*-Test (Unpaired) *p* Value
*Chrm2*	M_2_ muscarinic acetylcholine receptor	1.00	0.10	0.96	0.06	0.763
*Adra1a*	α_1A_ adrenoreceptor	1.00	0.44	0.49	0.08	0.318
*Adra1b*	α_1B_ adrenoreceptor	1.00	0.15	0.91	0.09	0.650
*Adra1d*	α_1D_ adrenoreceptor	1.00	0.24	0.19	0.08	0.035
*Adrb1*	β_1_ adrenoreceptor	1.00	0.02	0.98	0.19	0.928
*Adrb2*	β_2_ adrenoreceptor	1.00	0.38	1.09	0.15	0.830

**Table 7:** The mean gene fold changes underlying the cAMP pathway in *Pgc-1α^−/−^* compared to *WT* atria.

Gene	Protein	Mean WT Fold Change (*n* = 3)	WT SE	Mean *Pgc-1α*^−/−^ Fold Change (*n* = 3)	*Pgc*-*1α*^−/−^ SE	*t*-Test (Unpaired) *p* Value
*Adcy4*	Adenylyl cyclase type 4	1.00	0.08	0.64	0.05	0.017
*Adcy5*	Adenylyl cyclase type 5	1.00	0.38	0.83	0.15	0.706
*Pde2a*	cGMP-dependent 3′,5′-cyclic phosphodiesterase 2A	1.00	0.08	1.12	0.11	0.429
*Pde4d*	cAMP-specific 3′,5′-cyclic phosphodiesterase 4D	1.00	0.15	1.29	0.12	0.209
*Prkaca*	Catalytic subunit α of protein kinase A	1.00	0.14	0.85	0.09	0.394
*Prkar1a*	cAMP-dependent protein kinase type I-α regulatory subunit	1.00	0.11	1.47	0.14	0.061
*Prkar2a*	cAMP-dependent protein kinase type II-α regulatory subunit	1.00	0.05	0.81	0.10	0.161
*Prkar2b*	cAMP-dependent protein kinase type II-β regulatory subunit	1.00	0.37	1.78	0.37	0.212
*Camk2d*	Calcium/calmodulin-dependent protein kinase type II delta	1.00	0.07	1.09	0.14	0.585

**Table 8:** Other mean gene fold changes in *Pgc-1α^−/−^* compared to *WT* atria.

Gene	Protein	Mean WT Fold Change (*n* = 3)	WT SE	Mean *Pgc*-*1α*^−/−^ Fold Change (*n* = 3)	*Pgc*-*1α*^−/−^ SE	*t*-Test (Unpaired) *p* Value
*Tgfb1*	Transforming growth factor β	1.00	0.09	1.00	0.18	0.984
*Gjd3*	Gap junction delta-2 (GJD2)/connexin-36 (Cx36)	1.00	0.23	0.56	0.18	0.212
*Col1a1*	Collagen Type I α1 chain	1.00	0.13	0.84	0.21	0.545
*Col3a1*	Collagen Type III α1 Chain	1.00	0.43	0.74	0.30	0.640
*Tbx3*	T-box transcription factor	1.00	0.00	0.69	0.02	0.000
*Myh6*	Myosin heavy chain α isoform	1.00	0.14	0.76	0.16	0.322
*Nppa*	Natriuretic peptide A	1.00	0.10	2.19	1.01	0.308
*Trpc1*	Transient receptor potential channel 1	1.00	0.49	0.51	0.13	0.393
*Ppargc1a*	*PGC-1α*	1.00	0.10	0.00	0.00	0.000
*Ppargc1b*	*PGC-1β*	1.00	0.09	0.92	0.13	0.652

**Table 9:** Stratifications of mean gene fold changes in *Pgc-1α^−/−^* compared to *WT* atria.

**(A) Mean Gene Fold Changes in *Pgc-1α^−/−^* Compared to WT Atria in Ascending Order of *p* Value up to *p* ≤ 0.1.**						
**Gene**	**Protein**	**Mean WT Fold Change (*n* = 3)**	**WT SE**	**Mean *Pgc-1α^−/−^* Fold Change (*n* = 3)**	***Pgc-1α^−/−^*** **SE**	***t*-Test (Unpaired)** ***p*** **Value**
*Tbx3*	T-box transcription factor	1.00	0.00	0.69	0.02	0.000
*Cacna2d2*	Ca^2+^ channel, voltage-dependent, α_2_/δ_2_ subunit	1.00	0.05	0.50	0.07	0.005
*Scn5a*	Na^+^ voltage-gated channel type 5 subunit α, Na_v_1.5	1.00	0.11	0.52	0.04	0.012
*Adcy4*	Adenylyl cyclase type 4	1.00	0.08	0.64	0.05	0.017
*Hcn1*	Hyperpolarisation activated cyclic nucleotide-gated channel 1	1.00	0.07	0.50	0.12	0.022
*Atp1b1*	Na-K-transporting ATPase subunit β1	1.00	0.17	0.42	0.04	0.028
*Adra1d*	α-1D adrenoreceptor	1.00	0.24	0.19	0.08	0.035
*Casq2*	Calsequestrin 2	1.00	0.08	0.72	0.06	0.050
*Cacna1c*	Ca^2+^ channel, voltage-dependent, L type, α1C subunit (Cav1.2)	1.00	0.09	0.65	0.09	0.051
*Prkar1a*	cAMP-dependent protein kinase type I-α regulatory subunit	1.00	0.11	1.47	0.14	0.061
*Atp1a2*	Na-K-transporting ATPase subunit α2	1.00	0.12	0.46	0.17	0.062
*Atp1a1*	Na-K-transporting ATPase subunit α1	1.00	0.14	0.59	0.08	0.063
*Cacna2d1*	Ca^2+^ channel, voltage-dependent, α2/δ1 subunit	1.00	0.13	0.66	0.04	0.065
*Abcc9*	ATP-binding cassette (ABC) transporter subunit of ATP-sensitive K^+^ channels	1.00	0.11	1.65	0.24	0.071
*Abcc8*	ATP-binding cassette (ABC) transporter subunit of ATP-sensitive K^+^ channels	1.00	0.12	1.77	0.30	0.078
*Scn7a*	Na^+^ voltage-gated channel type 7 subunit α, Na_v_2.1	1.00	0.25	0.39	0.09	0.081
*Kcnn2*	intermediate/small K^+^ conductance Ca^2+^-activated channel, subfamily N, member 2 (KCa2.2)	1.00	0.30	0.33	0.03	0.090
*Hcn4*	Hyperpolarisation activated cyclic nucleotide-gated channel 4	1.00	0.09	0.46	0.24	0.100
**(B) Mean Gene Fold Changes in *Pgc-1α^−/−^* Compared to WT Atria Ordered by Size of Fold Change for Changes with *p* ≤ 0.1**						
**Gene**	**Protein**	**Mean WT Fold Change (*n* = 3)**	**WT SE**	**Mean *Pgc-1α^−/−^* Fold Change (*n* = 3)**	***Pgc-1α^−/−^*** **SE**	***t*-Test (Unpaired) *p* Value**
**Downregulated**						
*Adra1d*	α-1D adrenoreceptor	1.00	0.24	0.19	0.08	0.035
*Kcnn2*	intermediate/small K^+^ conductance Ca^2+^-activated channel, subfamily N, member 2 (KCa2.2)	1.00	0.30	0.33	0.03	0.090
*Scn7a*	Na^+^ voltage-gated channel type 7 subunit α, Na_v_2.1	1.00	0.25	0.39	0.09	0.081
*Atp1b1*	Na-K-transporting ATPase subunit β1	1.00	0.17	0.42	0.04	0.028
*Hcn4*	Hyperpolarisation activated cyclic nucleotide-gated channel 4	1.00	0.09	0.46	0.24	0.100
*Atp1a2*	Na-K-transporting ATPase subunit α2	1.00	0.12	0.46	0.17	0.062
*Cacna2d2*	Ca^2+^ channel, voltage-dependent, α_2_/δ_2_ subunit	1.00	0.05	0.50	0.07	0.005
*Hcn1*	Hyperpolarisation activated cyclic nucleotide-gated channel 1	1.00	0.07	0.50	0.12	0.022
*Scn5a*	Na+ voltage-gated channel type 5 subunit α, Na_v_1.5	1.00	0.11	0.52	0.04	0.012
*Atp1a1*	Na-K-transporting ATPase subunit α1	1.00	0.14	0.59	0.08	0.063
*Adcy4*	Adenylyl cyclase type 4	1.00	0.08	0.64	0.05	0.017
*Cacna1c*	Ca^2+^ channel, voltage-dependent, L type, α1C subunit (Ca_v_1.2)	1.00	0.09	0.65	0.09	0.051
*Cacna2d1*	Ca^2+^ channel, voltage-dependent, α2/δ1 subunit	1.00	0.13	0.66	0.04	0.065
*Tbx3*	T-box transcription factor	1.00	0.00	0.69	0.02	0.000
*Casq2*	Calsequestrin 2	1.00	0.08	0.72	0.06	0.050
**Upregulated**						
*Abcc8*	ATP-binding cassette (ABC) transporter subunit of ATP-sensitive K^+^ channels	1.00	0.12	1.77	0.30	0.078
*Abcc9*	ATP-binding cassette (ABC) transporter subunit of ATP-sensitive K^+^ channels	1.00	0.11	1.65	0.24	0.071
*Prkar1a*	cAMP-dependent protein kinase type I-α regulatory subunit	1.00	0.11	1.47	0.14	0.061
